# Assessment of pathogenesis of infective endocarditis by plasma IgG antibody titer test against periodontal bacteria

**DOI:** 10.1002/ccr3.1066

**Published:** 2017-08-17

**Authors:** Daichi Isoshima, Keisuke Yamashiro, Kazuyuki Matsunaga, Michitaka Shinobe, Nagako Nakanishi, Izumi Nakanishi, Kazuhiro Omori, Tadashi Yamamoto, Shogo Takashiba

**Affiliations:** ^1^ Department of Pathophysiology‐Periodontal Science Okayama University Graduate School of Medicine, Dentistry and Pharmaceutical Sciences 2‐5‐1 Shikata‐cho Kita‐ku Okayama 700‐8525 Japan; ^2^ Shinobe Clinic 5‐32‐2 Akatsutsumi Setagaya‐ku Tokyo 156‐0044 Japan; ^3^ Miyoshi Renal Clinic 3‐13‐2 Miyoshi‐cho Fuchu‐shi Tokyo 183‐0045 Japan; ^4^ Machidakeisen Hospital 2‐1‐47 Minami‐machida Machida‐shi Tokyo 194‐0005 Japan; ^5^ Department of Periodontics and Endodontics Okayama University Hospital 2‐5‐1 Shikata‐cho Kita‐ku Okayama 700‐8558 Japan

**Keywords:** bacteremia, IgG antibody titer test against periodontal bacteria, infective endocarditis, oral bacteria, periodontitis, *Porphyromonas gingivalis*

## Abstract

Oral bacteria cause infective endocarditis (IE), so severe periodontitis is thought to be high risk for IE. We suggest the identification of high‐risk patients by an IgG antibody titer test against periodontal bacteria might become common screening test.

## Introduction

Infective endocarditis (IE) is a cardiovascular disease caused by the inflammation of the inner tissues of the heart, the endocardium, usually of the valves 1. Bacteremia is essential for development of IE, and the main pathogens that cause IE are *Streptococcus* and *Staphylococcus* 2. A blood culture examination is usually performed to detect pathogenic bacteria because it is difficult to harvest infected heart tissue. However, the bacteria are often not detected because not enough of certain bacteria such as anaerobic periodontal bacteria stay alive for blood culture 3. Dental treatments, such as tooth extraction, periodontal treatment, and toothbrushing, are also known to cause bacteremia and IE 4. Some reports have shown that several oral bacteria are detected in extracted cardiovascular valves 5, 6, 7. Relapse of IE occurs frequently; therefore, appropriate oral hygiene and preoperative administration of antibiotics before dental treatment have been recommended 8.

Periodontitis is an infectious disease caused by oral bacterial infection, and severe periodontitis is thought to be high risk for IE 9; therefore, identification of high‐risk patients by common screening tests in both medical and dental services is necessary. A blood test for plasma IgG antibody titer against periodontal bacteria is one of the screening tests utilized to diagnose severe periodontitis in patients 10. Moreover, the usefulness of evaluation of IgG antibodies against periodontal pathogenic bacteria for prediction of vascular disease has already been reported 11.

In this study, we report a patient with severe periodontitis and relapse of IE, even though she received appropriate periodontal treatment. For the screening test, the patient's serum was taken for the plasma IgG antibody titer test against *Porphyromonas gingivalis* (Pg), and a remarkably high IgG titer was detected, which suggested severe periodontitis. Following cardiac surgery for IE, the existence of an oral microbial infection in cardiac tissue was confirmed because the genes of oral bacteria including Pg were detected using in vitro DNA amplification. We experienced the usefulness of this blood test to screen the patients with high risk for IE because of severe periodontitis.

## Case Report

### Patient information

The patient was a 69‐year‐old woman with a history of chronic glomerulonephritis with dialysis, hypertension, hypertensive retinopathy, and secondary hyperparathyroidism. Her general condition was controlled well, and there were no problems with her daily life and dialysis before her dental clinic visit.

**Figure 1:**
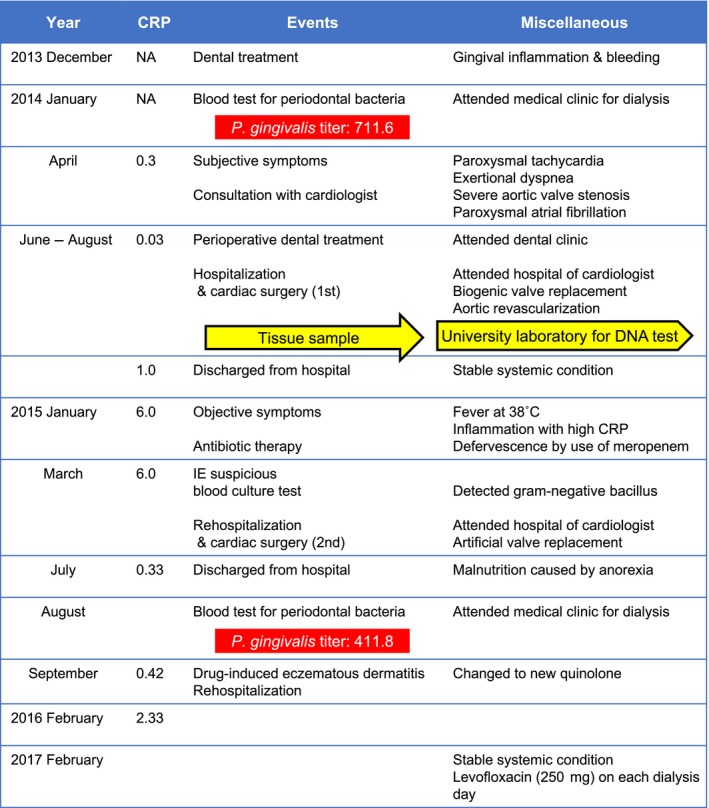
The summary of patient condition course. Patient condition course from December 2013 to February 2017 is summarized.

**Figure 2:**
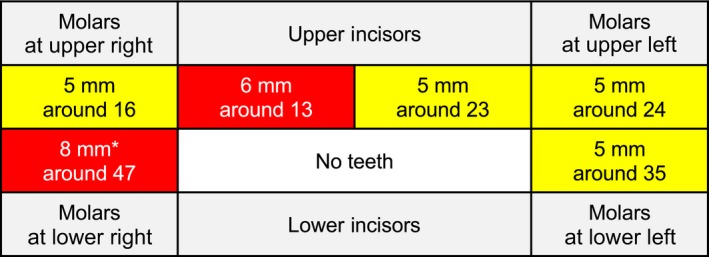
Periodontal condition. Dental record of basic periodontal examination was obtained from a private dental office. The patient has 21 present teeth, and the worst periodontal conditions in each of six segments are shown as representatives. Teeth are shown by codes defined by ISO system by the WHO. Segments with yellow show moderate periodontitis. Segments with red show severe periodontitis. *pus discharge and tooth mobility.

The summary of her condition course is shown in Figure 1. She had been receiving medical support and renal dialysis at the clinics of some of the authors. In December 2013, she visited a private dental clinic because of gingival bleeding and swelling and was diagnosed with moderate periodontitis (Fig. 2). However, there was a severe region on the right mandibular second molar (tooth number 47), around which bleeding and pus discharged from the periodontal pocket. At that time, one of the authors (attending physician) suspected experience periodontitis on glomerulonephritis and other medical conditions. Thus, he tested a plasma IgG antibody titer against periodontal bacteria as shown below.

In April 2014, the patient experienced paroxysmal tachycardia and exertional dyspnea; therefore, she was referred to the cardiovascular hospital and diagnosed with severe aortic valve stenosis and paroxysmal atrial fibrillation. During the perioperative period, she received periodontal treatment, followed by biogenic valve replacement surgery and aortic revascularization. After the surgery, formalin‐fixed and paraffin‐embedded pathological specimen piece of the removed valve was sent to Okayama University Hospital for DNA detection of oral bacteria.

### Plasma IgG antibody titer test against periodontal bacteria

We used the DEMECAL® blood examination system (Leisure, Inc., Tokyo, Japan) to measure the titers of IgG antibodies to periodontal bacteria such as Pg, *Aggregatibacter actinomycetemcomitans* (Aa), *Prevotella intermedia* (Pi), and *Eichenerra corrodens* (Ec) in the patient's blood according to the manufacturer's instructions 10. We performed this test twice at an author's clinic, once after her first dental clinic visit (January 2014) and again after her valve replacement surgery (August 2015).

### Bacteria DNA detection by PCR and sequencing from extracted cardiac valve

Bacterial DNA was extracted from the specimen piece using phenol–chloroform 12. We constructed specific primers according to 16S rRNA genes for Pg, Aa, Pi, Ec, *Fusobacterium nucleatum* (Fn), *Streptococcus mutans* (Sm), *Staphylococcus aureus* (Sa), and universal primers for nested PCR (Table 1). We performed polymerase chain reaction (PCR) using the Veriti Thermal Cycler (Applied Biosystems, Foster City, CA, USA) as described previously 13.

**Table 1:** Primer design for each bacterial species

Bacteria	Forward primer	Reverse primer	Product size
*Aa*	CAAGTGTGATTAGGTAGTTGGTGGG	GATTTCACACCTCACTTAAAGGTCC	376 bp
*Pg*	CTTGAGTTCAGCGGCGGCAG	AGGGAAGACGGTTTTCACCA	378 bp
*Pi*	AATACCCGATGTTGTCCACA	TTAGCCGGTCCTTATTCGAA	339 bp
*Ec*	CTAATACCGCATACGTCCTAAG	CTACTAAGCAATCAAGTTGCCC	668 bp
*Sm*	GGTCAGGAAAGTCTGGAGTAAAAGGCTA	GCGGTAGCTCCGGCACTAAGCC	282 bp
*Fn*	AGAGTTTGATCCTGGCTCAG	GTCATCGTGCACACAGAATTGCTG	360 bp
*Sa*	CTGGAACTGAGACACGGTCC	GTTTACGGCGTGGACTACCA	502 bp
16S universal primer	AGAGTTTGATCCTGGCTCAG (27 Forward)	GGTGTGTACAAGGCCCGGGAACG (1378 Reverse)	1351 bp
16S universal primer	AGAGTTTGATCCTGGCTCAG (27 Forward)	ACGTCRTCCMCACCTTCCTC (1074 Reverse)	1047 bp

Briefly, PCR was performed under the following conditions: 95°C for 10 min, followed by 35 cycles at 95°C for 30 sec, 60°C for 30 sec, 72°C for 1 min, and final extension at 72°C for 4 min. After PCR, the reactions were kept at 4°C overnight and then frozen at −20°C. For Sm, Fn, Sa, Pi, and Ec detection, the first PCR was performed with 1 μL of template DNA solution, 1 μL of forward and reverse primers (10 pmol/μL), and 25 μL of AmpliTaq Gold® 360 Master Mix (Applied Biosystems) with a final volume of 50 μL. The second PCR was performed with 1 μL of the PCR product from the first PCR and under the same conditions as the first PCR. For Aa and Pg detection, the first PCR was performed with 1 μL of template DNA, 1 μL each of universal primers (27 Forward and 1378 Reverse; 10 pmol/μL), and 9.25 μL of TaKaRa Ex Taq® (Takara Bio, Tokyo, Japan) with a final volume of 50 μL. The second PCR was performed with 1 μL of the PCR product from the first PCR and 1 μL each of universal primers (27 Forward and 1074 Reverse; 10 pmol/μL) under the same conditions as the first PCR. The third PCR was performed with 1 μL of PCR product from the second PCR and bacteria‐specific primers for Aa or Pg under the same conditions as the first PCR. DNA extracted from each cultured bacterium (Aa, Pg, Pi, Ec, Sm, Fn, and Sa) was used for positive control. We confirmed the specific bands for each set of primers by agarose gel electrophoresis.

For further confirmation, we extracted the band areas from the agarose gel using the QIAEX II® Gel Extraction Kit (Qiagen, Hilden, Germany) and performed DNA sequencing using ABI PRISM® 310 Genetic Analyzer (Applied Biosystems) to examine homology search using GenBank (https://www.ncbi.nlm.nih.gov/genbank/) for each bacterium.

### Ethical consideration

This case study was conducted according to the guidelines of Okayama University Hospital, and written informed consent was obtained from the patient.

## Results

IgG titers against Aa, Pg, Pi, and Ec were measured by the manufacturer's laboratory. Only Pg IgG antibody was elevated to an extremely remarkable degree. After the first dental treatment, the IgG titer against Pg was >700 (Table 2). After cardiac surgery, her condition was stable for a while. In January 2015, there were no obvious signs of infection, but she had a fever (about 38°C, CRP = 6, WBC = 10,000 cells/μL). In order to prevent bacterial infection, she was administered with antibacterial medication (meropenem) continuously.

**Table 2:** Plasma IgG antibody titer test values against oral bacteria

Bacteria	January 2014	August 2015
Aa	0	0.6
Pg	711.6	411.8
Pi	−0.6	−0.5
Ec	−0.5	−0.5

In March 2015, the patient revisited the cardiovascular hospital because she experienced severe exertional dyspnea and fatigue. She was diagnosed with IE and subvalvular abscess. Thereafter, she was hospitalized and underwent valve replacement surgery again. After surgery, the patient's hospitalization was prolonged because she still had a slight fever. At that time, the plasma IgG antibody titer against Pg had decreased but remained at high level of >400 (Table 2). Unfortunately, further blood tests were not performed because the commercial service for periodontal bacteria testing was discontinued.

**Figure 3:**
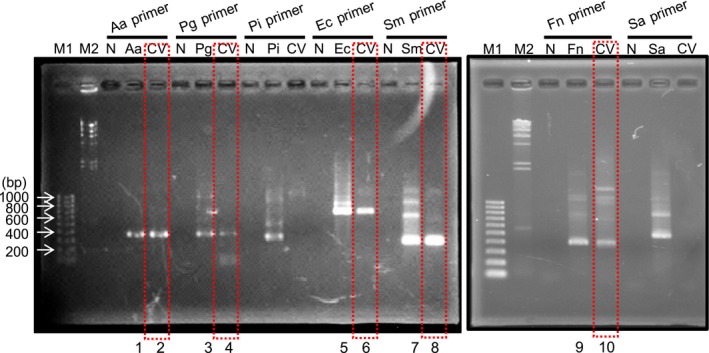
Detection of bacterial DNA by PCR. Amplified DNA was electrophoresed on 0.7% agarose gel and visualized by ethidium bromide staining. Bacterial DNA in cardiac valve (CV) was detected. Square with red dashed line shows detected bacteria from CV samples. DNA bands in numbered lanes (1–10) were extracted and used for DNA sequencing for homology search (Table 3). M1: DNA marker (100‐bp ladder); M2: DNA marker (*λ* DNA digested by *Hin*d III); N: negative control without bacterial DNA; Aa (*Aggregatibacter actinomycetemcomitans*), Pg (*Porphyromonas gingivalis*), Pi (*Prevotella intermedia*), Ec (*Eichenerra corrodens*), Sm (*Streptococcus mutans*), Fn (*Fusobacterium nucleatum*), and Sa (*Staphylococcus aureus*): positive controls with appropriate bacterial DNA; CV: test DNA sample extracted from cardiac valve obtained at her first cardiovascular surgery.

Several oral bacteria such as Aa, Pg, Ec, Sm, and Fn were detected from the specimen by PCR (Fig. 3), and we confirmed that the PCR products had high homology for each bacterium by homology search (Table 3). The PCR products of the test samples had high homology of >90% as positive control for each bacterium (sample numbers 1, 3, 5, 7, and 9 shown at the bottom of the gel; Fig. 3).

**Table 3:** Homology search for PCR products

DNA samples	Primers	Detected DNA	Homology (%)
1 Aa control	Aa primer	*Aggregatibacter actinomycetemcomitans*	100
2 CV sample	Aa primer	*Aggregatibacter actinomycetemcomitans*	99
3 Pg control	Pg primer	*Porphyromonas gingivalis*	100
4 CV sample	Pg primer	*Porphyromonas gingivalis*	100
5 Ec control	Ec primer	*Eikenella corrodens*	100
6 CV sample	Ec primer	*Eikenella corrodens*	99
7 Sm control	Sm primer	*Streptococcus mutans*	96
8 CV sample	Sm primer	*Streptococcus mutans*	98
9 Fn control	Fn primer	*Fusobacterium nucleatum*	100
10 CV sample	Fn primer	*Fusobacterium nucleatum*	94

## Discussion

IE is a disease that forms vegetation containing bacteria in valves, the endocardium, and inner membrane of large blood vessels and is associated with various symptoms such as bacteremia, embolization, and cardiac disorder 14. IE is not a highly prevalent disease, but it can be a lethal disease and induce various complications once it develops 15. The most important aspects to consider prior to IE treatment are (1) appropriate prevention for high‐risk patients, (2) appropriate diagnosis, (3) effective antibacterial drug choice, (4) early detection of complications, and (5) timely cardiovascular surgery 8. Dental treatments such as tooth extraction, root canal treatment, and periodontal treatment can sometimes cause transient bacteremia. It is thought that bacteria mainly invade blood vessels from the oral cavity 16. Thus, it is widely understood that dental treatment is one of the high‐risk procedures for developing IE 17. In the United Kingdom (UK), guidelines from the National Institute for Health and Clinical Excellence (NICE) in 2008 recommended complete cessation of antibiotic prophylaxis for prevention of IE before invasive dental procedures. However, the incidence of infective endocarditis has increased significantly in the UK since the introduction of the guidelines 18. It is reported that gram‐positive bacteria such as *Streptococcus viridians*,* Streptococcus bovis*, and *Staphylococcus aureus* are mainly pathogenic bacteria for IE. However, fungi and HACEK organisms such as *Haemophilus* species, *Aggregatibacter* species, *Cardiobacterium hominis*,* Eikenella corrodens*, and *Kingella* species are low‐pathogenic gram‐negative bacteria that are also detected by blood culture examination 19. Considering these findings, there is no doubt that some oral bacteria are involved in IE development.

Periodontitis is caused by infection by bacteria such as Pg, Aa, Pi, and *Tannerella forsythia* 20. Once periodontitis progresses, it is thought that bacteria in the gingival pocket invade blood vessels and induce bacteremia, especially in patients who have multiple health problems such as diabetes, steroid use, and chronic kidney disease. In this report, as the patient had chronic glomerulonephritis with dialysis, it is reasonable to assume she is at potential risk to developing bacteremia by periodontitis. Pg is a major periodontal bacterium with strong pathogenicity for periodontitis. As Pg is a gram‐negative and anaerobic bacterium, it is difficult to detect by a regular blood culture test. Instead of a culture test, it is reported that Pg DNA was detected in a cardiac valve by PCR 6. It has also been reported that Pg is involved in intimal hyperplasia in the aorta 21. Therefore, infection of Pg must be considered as a high‐risk event for IE. In this case, the patient's plasma IgG antibody titer against Pg was remarkably high (>700 titers as shown in Table 1) compared to severe periodontitis patients reported previously 10. Her chronic periodontitis was mostly moderate but partially severe, resulting from remarkably severe infection of Pg and significantly high immune response against Pg. In addition to Pg, Aa, Ec, Sm, and Fn were detected by PCR from the cardiac valve specimen; thus, it is clear that oral bacteria, especially periodontal pathogens, must be related to IE in this case. For IE prevention, it is important to identify severe periodontal patients before dental treatment by examination and performing appropriate procedures such as antibacterial drug administration. However, basic medical tests do not identify oral infections such as periodontitis; therefore, most physicians cannot identify patients with severe periodontitis. To prevent IE related to oral bacterial infection, regular medical tests that are easy to perform, minimally invasive, and taken in various locations are anticipated.

Blood test for IgG antibody titer against oral bacteria using plasma or sera can be potentially used as a regular test to detect oral bacterial infection. In this report, we used the DEMECAL® blood examination system, modified for detection of periodontal bacteria. This test needs blood sampling from a fingertip, but it may be convenient for patients because it is self‐operated and a mail‐medicine system 22. This system is very easily performed, minimally invasive, and taken in various locations. Indeed, we succeeded in detecting high‐risk condition for IE using this test. However, the plasma IgG antibody titer test against periodontal bacteria includes some problems regarding sensitivity and specificity. Although we reported sensitivity and specificity of 0.774 and 0.586, respectively 10, the findings are not sufficient enough for wide use of the test. We have selected highly sensitive antigenic proteins of Pg and are developing a high‐speed automated test system (manuscript under preparation for submission). The sensitivity was remarkably improved; therefore, the newly designed test will be more useful in the near future. It was recently reported that Pg in combination with *Tannerella forsythia* was associated with moderate‐to‐severe periodontitis with an odds ratio 23. Further clinical studies including prospective and retrospective observations are needed to develop a highly sensitive examination.

## Conclusion

In this report, we identified a patient who developed IE, possibly related to oral bacterial infection, using an IgG antibody titer test against periodontal bacteria. The infection was confirmed by the detection of bacterial DNA. This suggested that IgG antibody titer test could be used for early screening of high‐risk patients developing IE.

## Authorship

DI: performed PCR analysis and involved in study conception. KY: drafted the manuscript. KM: performed PCR analysis. MS: was attending physician and involved in study conception and design. NN: was attending physician. IN: was attending physician. KO: made critical revision. TY: made critical revision. ST: made critical revisions and is the corresponding author.

## Conflict of interest

The authors have no conflict of interests to disclose.
